# Gut Microbiota Play an Essential Role in the Antidiabetic Effects of Rhein

**DOI:** 10.1155/2018/6093282

**Published:** 2018-07-09

**Authors:** Ruifeng Wang, Pu Zang, Junxiu Chen, Fei Wu, Zhouqin Zheng, Jian Ma, Cuihua Yang, Hong Du

**Affiliations:** ^1^Department of Endocrinology, Jinling Hospital, Nanjing, China; ^2^Medical School of Nanjing University, Nanjing, China; ^3^Southern Medical University, Guangzhou, China

## Abstract

It is clear that the gut microbiota can affect host metabolism and alterations of the gut microbiota can link with metabolic disease. Rhein has been used in traditional Chinese medicine with putative antidiabetic effects. Here we show that oral administration of rhein for 6 weeks can significantly reduce fasting blood glucose (FBG) level (8.30 ± 4.52 mmol/l versus 18.89 ± 6.06 mmol/l,* p* < 0.01), elevate the active glucagon-like peptide 1 (GLP-1) level (22.21 ± 2.61 pmol/l verss 14.46 ± 5.22 pmol/l,* p* < 0.05), and increase the number of L-cells in the terminal ileum. The antidiabetic effect of rhein is abrogated in db/db mice treated with rhein in combination with broad-spectrum antibiotics. We observed that the abundance of the Bacteroidetes is increased in mice treated with rhein (0.361±0.022 versus 0.185 ± 0.055,* p* < 0.05,). In addition, there is no significant difference in diversity between rhein-treated groups and the controls (Shannon index:* p* = 0.88; Simpson index:* p* = 0.86). Taken together, our results indicate that modulation of the gut microbiota may play an essential role in the antidiabetic effects of rhein.

## 1. Introduction

The prevalence of type 2 diabetes is increasing rapidly and it has become a worldwide health problem. It is now clear that the gut microbiota can affect host metabolism and alterations of the gut microbiota can link with metabolic disease [1]. The gut microbiota consists of approximately 1,000 to 1,500 different bacterial species, containing at least 100 times more genes than the genome encoded by the human genome. The microbiome provides metabolic functions that the host does not have to develop itself. Furthermore, the interaction between the nutritional content of the diet and bacterial metabolism in the gut produces a metabolic footprint with an extensive number of bioactive metabolites that can influence the host [2]. The human metagenome-wide association study (MGWAS) demonstrates that concentrations of butyrate-producing bacteria such as* Roseburia intestinalis* and* Faecalibacterium prausnitzii* are decreased in T2DM subjects [3]. The enteroendocrine cells distributed in the epithelial lining secrete a vast number of peptides with profound effects on host physiology. Glucagon-like peptide 1 (GLP1) and peptide YY (PYY) are the most studied peptides [4]. Both of them are secreted by L-cells that are most abundant in the distal small intestine and have several biological functions in host physiology ranging from controlling appetite and regulating stomach emptying and gut transit to acting as incretin hormones and promoting *β*-cell survival and proliferation [4]. The nondigestible carbohydrates modulate the gut microbiota and improve glucose and energy homeostasis by fermentation. Several nondigestible carbohydrates are fermented by the gut microbiota. The major products of nondigestible carbohydrate metabolism by the gut microbiota are short-chain fatty acids (SCFA; e.g., acetate, propionate, butyrate, lactate, and succinate), which can stimulate gut peptide production. The SCFA produced in the intestine by bacterial fermentation of undigested carbohydrates stimulates GLP-1 and PYY secretion via GPCRs [5]. Rhein, an anthraquinone compound extracted from rhubarb, has been used in traditional Chinese medicine with putative antidiabetic effects for more than 2,000 years. Our research team and others have demonstrated that rhein can improve glucose tolerance in diabetic mice, and its hypoglycemic effect is even stronger than rosiglitazone [6]. Rhein protects pancreatic *β*-cells from dynamin-related protein-1-mediated mitochondrial fission and cell apoptosis under hyperglycemia [6]. Although oral administration of rhein can significantly reduce fasting blood glucose, intravenous application of rhubarb injection had no hypoglycemic effect on experimental animals.

These findings raise the possibility that gut microbiota play an essential role in the antidiabetic effects of rhein. In this study, we focus on how rhein regulates gut microbiota and how it contributes to the enteroendocrine function.

## 2. Materials and Methods

### 2.1. Animals

C57BL/KsJ-db/db mice were provided by the Research Institute of Nephrology, Jinling Hospital, Nanjing University School of Medicine, China. The study followed the Chinese community's regulations for animal experiments and was approved by Animal Care and Use Committee of Jinling Hospital, Nanjing. Six-week-old C57BL/KsJ-db/db mice (*n* = 32), weighing 32.6±2.4 g, were housed 8 mice/cage in the specific-pathogen-free environment (12 h light cycle).

### 2.2. Materials and Research Design

All mice were fed with normal-chow diet and had free access to water. After one week of acclimatization, the mice were randomly divided into four groups (*n* = 8):

1. Control group (Con) treated with 1% natrium cellulose solution (Sigma, USA) as placebo
2. Rhein group (Rh) treated with rhein (120 mg/kg/day, Nanjing Tisiaime Institute of Traditional Chinese Medicine; purity > 99%)
3. Antibiotic group (Anti) treated with broad-spectrum antibiotics (Vancomycin 10 mg/kg/day, Carbenicillin 50 mg/kg/day, Metronidazole 50 mg/kg/day, and Neomycin 30 mg/kg/day, Sigma, USA)
4. Rhein and antibiotic combined group (Rh-Anti) treated with rhein and broad-spectrum antibiotics (rhein 120 mg/kg/day and Vancomycin 10 mg/kg/day, Carbenicillin 50 mg/kg/day, Metronidazole 50 mg/kg/day, and Neomycin 30 mg/kg/day)

Food intake (on per cage basis), fasting blood glucose, and body weights were recorded once a week. Intraperitoneal glucose tolerance test was performed after 6-week treatment. Fasting blood glucose and body weights were recorded once a week during the study and an intraperitoneal glucose tolerance test was performed at the end of the experiment. Feces were collected at three different time points during the study (0, 3, and 6 weeks). Mice were picked up so as to collect the feces as soon as they defecated and they were stored at -80°C immediately until analysis. After 6 weeks of treatment, mice in each group were sacrificed. At the time of sacrifice, mice were fasted for 10 hours and thereafter anesthetized with an intraperitoneal injection of pentobarbital (Dormicum, Hoffman-La Roche, Basel, Switzerland). Blood was drawn by atrial puncture followed by cervical dislocation and collected in centrifuge tubes containing DPP-IV inhibitor (Millipore, USA, NM_001935.3). Terminal ileum (2-3 mm) was collected and put in buffer containing paraformaldehyde or liquid nitrogen immediately. All of the above operations were performed on ice. The active GLP-1 was assessed by ELISA Kit (Millipore, USA, EGLP-35K) according to the manufacturer's instructions; L-cell in terminal ileum was stained by GLP-1 antibody (Abcam, UK, ab22625).

### 2.3. DNA Extraction and Sequencing

Total genome DNA from samples was extracted using CTAB/SDS method. DNA concentration and purity were monitored on 1% agarose gels. According to the concentration, DNA was diluted to 1 ng/*μ*L using sterile water. 16S rRNA genes of distinct regions (16SV4/16SV3/16SV3-V4/16SV4-V5) were amplified using specific primer (e.g., 16S V4: 515F-806R) with the barcode. All PCR reactions were carried out with Phusion® High-Fidelity PCR Master Mix (New England Biolabs). Mix 1X loading buffer (containing SYB green) with the same volume of PCR products and operate electrophoresis on 2% agarose gel for detection. Samples with bright main strip between 400 and 450 bp were chosen for further experiments. PCR products were mixed in equidensity ratios. Then, mixed PCR products were purified with Qiagen Gel Extraction Kit (Qiagen, Germany). Sequencing libraries were generated using TruSeq® DNA PCR-Free Sample Preparation Kit (Illumina, USA) following the manufacturer's recommendations and index codes were added. The library quality was assessed on the Qubit@ 2.0 Fluorometer (Thermo Scientific) and Agilent Bioanalyzer 2100 system. Finally, the library was sequenced on an Illumina HiSeq 2500 platform and 250 bp paired-end reads were generated.

### 2.4. Sequence Analysis and Species Annotation

Sequences analysis was performed by Uparse software (v7.0.1001). Sequences with >97% similarity were assigned to the same OTUs. A representative sequence for each OTU was screened for further annotation. For each representative sequence, the Greengenes Database was used based on RDP 2 classifier (version 2.2) algorithm to annotate taxonomic information.

### 2.5. Statistical Analysis

The data are presented as the mean ± standard error. For all comparisons,* p* values < 0.05 were considered as statistically significant, assessed using Student's *t*-test (for paired samples) or the ANOVA test (for more than two groups) by SPSS. The gut microbiota structure and composition were assessed by quantifying and interpreting similarities based on intra- and intergroup diversity analyses (alpha and beta diversity, resp.). For alpha diversity, Shannon and Simpson's diversity indices were calculated. Both indices take evenness and species richness into account but the Simpson index is more weighted towards the abundance of the most common species than species richness. Beta diversity was assessed using phylogeny-based generalized UniFrac distances (with the parameter controlling weight on abundant lineages = 0.5) calculated with the GUniFrac package of R. For this, we first reduced the alignment and the OTU table to one representative sequence per OTU and then obtained a distance matrix from uncorrected pairwise distances between aligned sequences, and finally constructed a relaxed neighbor-joining phylogenetic tree using mothur and Clearcut. Comparisons among groups of participants were performed using the adonis function (ANOVA using all the indices in our studies were calculated with QIIME (version 1.7.0) and displayed with R software (version 2.15.3)). In order to identify the specific bacterial phylotypes that were altered by rhein, we performed the LEfSe analysis. We were particularly interested in OTUs displaying strong associations in the LDA (represented by OTUs with [log10] LDA scores >4). We used linear discriminant analysis (LDA) effect size (LEfSe) to agnostically identify microbial biomarkers [7]. LEfSe uses the nonparametric factorial Kruskal-Wallis sum-rank test to detect individual OTUs with significant differential abundance among groups of participants and then performs a set of pairwise tests among groups of participants using the unpaired Wilcoxon rank-sum test and finally uses LDA to estimate the effect size of each differentially abundant OTU. The strength of LEfSe compared with standard statistical approaches is that, in addition to providing* p* values, it provides an estimation of the magnitude of the association between each OTU and the grouping categories (e.g., Rh and Con) through the LDA score. For stringency, microbial biomarkers in our study were retained if they had a* p* > 0.05 and a (log10) LDA score > 4.

## 3. Results

### 3.1. Rhein Improves Glucose Homeostasis

**Figure 1:**
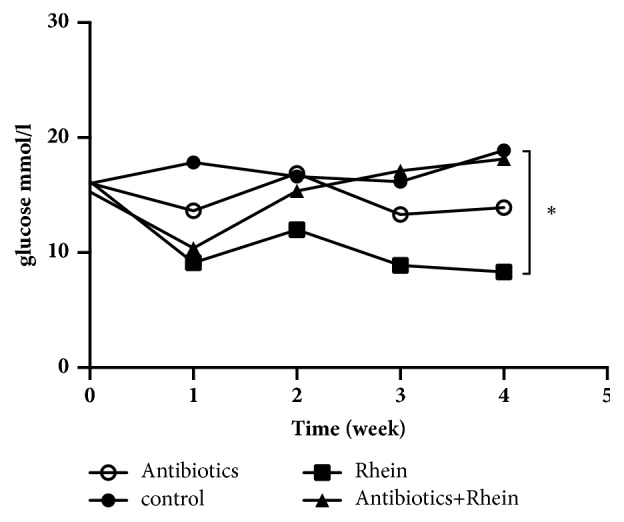
**Glucose homeostasis in db/db mice**. Fasting blood glucose (FBG) level during the study. Data are presented as means with standard error of the mean (SEM).

Oral administration of rhein for 4 weeks significantly reduced fasting blood glucose (FBG) level (8.30±4.52 mmol/l versus 18.89 ±6.06 mmol/l,* p* < 0.01) in db/db mice compared with the control group (Figure 1). However, it was shown that the FBG of db/db mice treated with rhein + antibiotics had no significant difference versus the control mice (18.14±3.34 mmol/l versus 18.80±6.07 mmol/l,* p* > 0.05), suggesting that the antibiotics weaken the hypoglycemic effect of rhein (Figure 1).

### 3.2. Rhein Treatment Increases the Plasma Active GLP-1 and the Number of L-Cells

**Figure 2:**
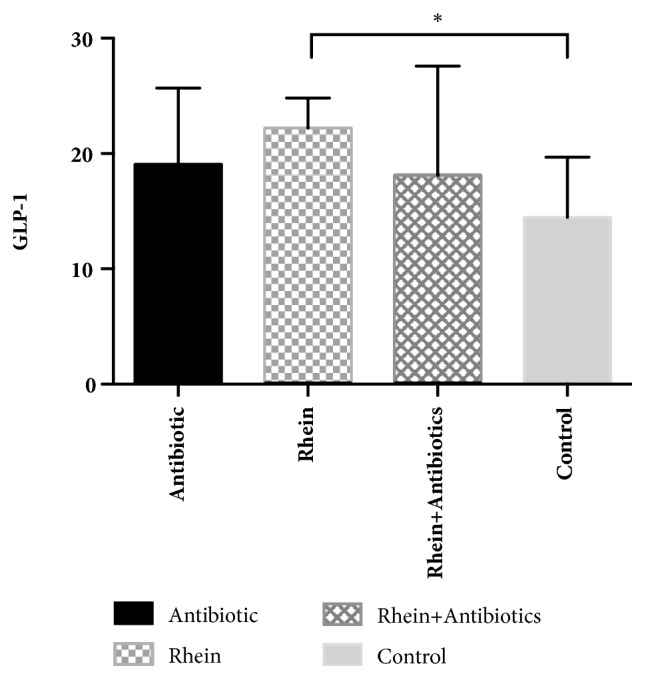
**Plasma active GLP-1 (7–36) and GLP-1 gene expression in db/db mice**. Data are presented as means with standard error of the mean (SEM).

**Figure 3:**
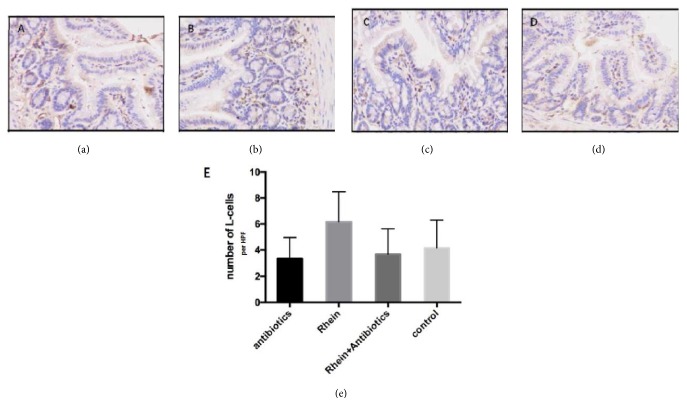
**L-cell immunohistochemical staining in db/db mice**. (a) Rhein-treated group, (b) control group, (c) antibiotics-treated group, and (d) rhein + antibiotics-treated group (magnification: 200x). Brown granules on behalf of GLP-1-positive staining. (e) The number of L-cells per HPF in the terminal ileum.

Plasma active GLP-1 (7–36) in the rhein-treated group was significantly higher compared with the control group (22.21 ± 2.61 pmol/l versus 14.46 ± 5.22 pmol/l,* p* < 0.05). There were no significant differences in the other three groups (Figure 2). It was shown that plasma active GLP-1 had no significant increase in mice treated with antibiotics or rhein + antibiotics compared with the control group (Figure 2). L-cell immunohistochemical staining in the terminal ileum was performed. GLP-1 immunoreactive cells were easily identified by their classical bottle-like shape (Figure 3). The mean and SD per high-power field (X400) of cells stained with anti-GLP-1 of the 4 groups are 3.4±1.8, 6.3±2.7, 3.7±2.0, and 4.5±2.3, respectively. The total number of L-cells was higher in rhein-treated mice.

### 3.3. Rhein Treatment Altered Gut Microbiota of Diabetic Mice

**Figure 4:**
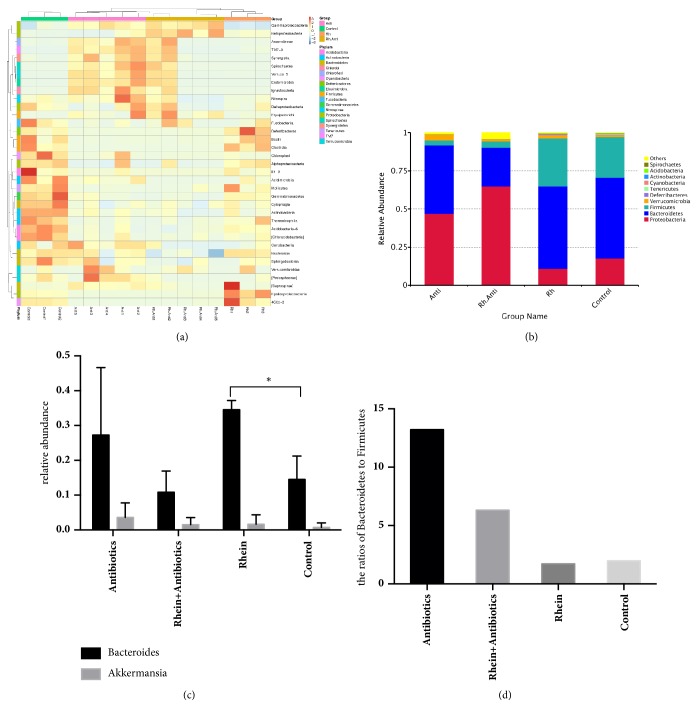
**Composition of gut microbiota in db/db mice**. (a) Heat map of the relative abundance of gut microbiota composition in db/db mice in the level of phylum. (b) The relative abundance of top 10 species in each group in the level of phylum. (c) The relative abundance of* Bacteroides* and* Akkermansia* in each group. (d) The ratios of Bacteroidetes to Firmicutes.

Since the composition of the gut microbiota is associated with T2DM, we investigated the effect of rhein on the composition of the gut microbiota. According to the species annotation and abundance of all the samples in the level of class, we selected the top 35 classes and drew them into the heat map (Figure 4(a)). The relative abundance of different species is displayed in Figure 4(b). The relative abundance of* Bacteroides* in the rhein-treated group significantly increased compared with the control group (mean 0.361 and 0.185,* p* = 0.02) and* Akkermansia* was mildly increased as well (mean 0.0214 and 0.010,* p* = 0.673) (Figure 4(c)). Rhein treatment decreased the ratio of Bacteroidetes and Firmicutes compared with the control group (1.712 and 1.987) (Figure 4(d)). Furthermore, antibiotics had major effects on the gut microbiota communities. Mice treated with antibiotics (both in the Anti group and in the Rh-Anti group) had a significantly higher relative abundance of proteobacteria than the other groups (0.471 and 0.177,* p* = 0.02; 0.651 and 0.177,* p* = 0.004, resp.) (Figure 4(b)). The gut flora in mice treated with rhein + antibiotics was similar to the mice treated with antibiotics alone (Figure 4(b)). These results imply that alterations of gut microbiota contribute to the antidiabetic effect of rhein.

**Figure 5:**
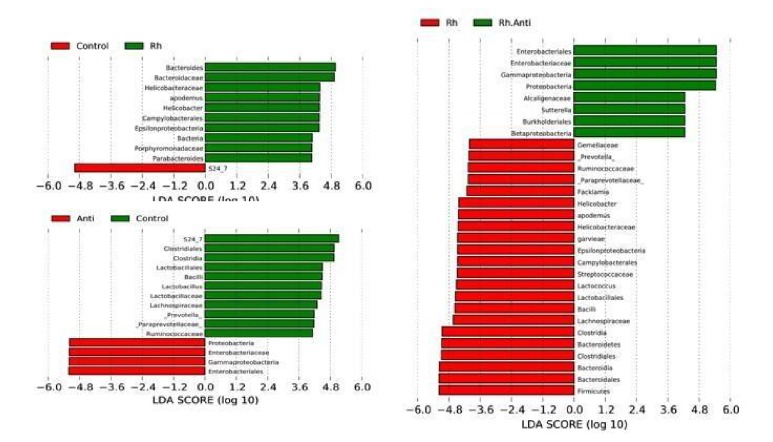
LDA scores (log10) of the OTUs displaying differences between pairs of groups.

Compared with the control group, rhein treatment significantly altered several OTUs: OTUs belonging to* Bacteroides*, Bacteroidaceae, Helicobacteraceae,* Apodemus*,* Helicobacter*, Campylobacterales, Epsilonproteobacteria, Bacteria, Porphyromonadaceae, and* Parabacteroides* were overrepresented in the rhein-treated group. Notably, many of those are negatively correlated with obesity such as* Bacteroides* and* Parabacteroides* [8]. Antibiotics also had great effects on the gut flora. OTUs of Proteobacteria, Enterobacteriaceae, Gammaproteobacteria, and Enterobacteriales increased in mice treated with antibiotics compared with controls. Additionally,* Bacteroides*, which significantly increased in the rhein-treated group, was decreased in the mice treated with antibiotics and rhein + antibiotics (Figure 5).

### 3.4. Rhein Treatment Maintains the Diversity of gut Microbiota

**Figure 6:**
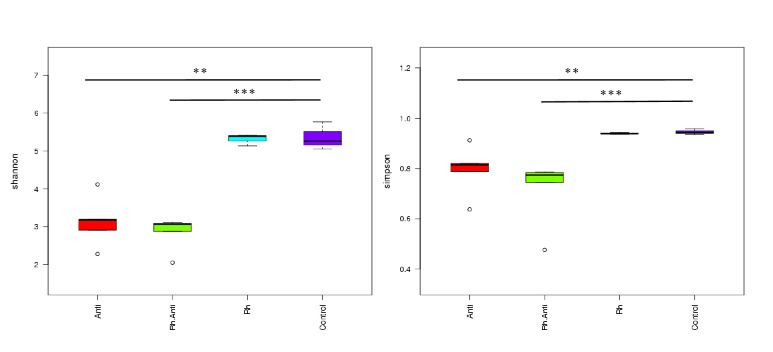
**Diversity of the microbiota in feces**. The area of each peak expressed as the proportion of the total area. *∗* indicates* p* < 0.05; *∗∗* indicates* p* < 0.01; *∗∗∗* indicates* p* < 0.001.

We showed the diversity within the four groups. Alpha diversity is applied in analyzing the complexity of species diversity for each group through 2 indices: Shannon and Simpson (Figure 3). It was shown that there was no difference in Shannon (H') and Simpson's diversity index (D) between rhein-treated groups and the controls (Shannon_Wilcoxon: D = -0.33,* p* = 0.88; Simpson_Wilcoxon: D = 0.33,* p* = 0.86), suggesting that rhein has no effect on the diversity of the gut microbiota. However, groups treated with antibiotics (in both Anti and Rh-Anti groups) showed a significant decline in the diversity of microbiota compared with the control group (Shannon_Wilcoxon: D = -6.73,* p* = 0.005; D = 8.93,* p* = 0.0006; Simpson_Wilcoxon: D = 9.86,* p* = 0.0001; D = -6.13,* p* = 0.004, resp.) and the Rh group (Shannon_Wilcoxon: D = -7.06,* p* = 0.003; D = 9.26,* p* = 0.0005; Simpson_Wilcoxon: D = -6.13,* p* = 0.004; D = 9.53,* p* = 0.0002, resp.). The Box-plot on the left was calculated by the Shannon index and the right one was calculated by the Simpson index (Figure 6).

## 4. Discussion

In this study, we demonstrate that treatment with rhein significantly improves glucose tolerance. The hypoglycemic effect of rhein is greatly abrogated without the healthy gut microbiota. The mechanism of the antidiabetic effect of rhein has not yet been fully elucidated. Here, we studied the interaction between the hypoglycemic effect of rhein and gut microbiota.

In this study, mice treated with rhein had lower FBG than the controls. However, the FBG of db/db mice treated with rhein + antibiotics showed no decline. The hypoglycemic effect of rhein is significantly receded by the antibiotics, indicating that gut microbiota play a key role in the antidiabetic effect of rhein. It was reported previously that intravenous application of rhubarb injection has no hypoglycemic effect in experimental animals [9]. Our results are inconsistent with the previous studies of the metabolic effects of rhein. Our study shows that rhein modulates gut microbiota and reduces the FBG of db/db mice without affecting the diversity of the community.

Our study demonstrates that rhein treatment increases the abundance of* Bacteroides* and* Akkermansia*. A large number of reports were concerned with the gut microbiota in mouse models of obesity, with most of the results highlighting an increase in Firmicutes and a decrease in Bacteroidetes associated with obesity [10, 11]. When obese humans were put on either a fat-restricted or carbohydrate-restricted low-calorie diet, an increase in the abundance of Bacteroidetes and a decrease in Firmicutes were seen [12]. Rhein treatment also decreases the ratio of Bacteroidetes and Firmicutes. The ratios of Bacteroidetes to Firmicutes have a significant positive correlation with the plasma glucose concentration [13].* Akkermansia muciniphila* has been identified as mucin-degrading bacterium that resides in the mucus layer. It may represent 3–5% of the microbial community in healthy subjects [14, 15], and its abundance negatively correlates with body weight in type 1 diabetes [16, 17]. A recent metagenomic study found that some of the genes belonging to* Akkermansia muciniphila* are enriched in type 2 diabetic subjects and it is the dominant human bacterium that abundantly colonizes this nutrient-rich environment. Hansen et al. reported a decrease in diabetes incidence in NOD mice treated with vancomycin from birth to 28 days of age and predominance of* Akkermansia muciniphila* bacteria in treated mice. This also demonstrates that gut microbiota play an important role in the antidiabetic effect of metformin through their impact on the proliferation of* Akkermansia* [18].

In this study, rhein treatment had no effect on the diversity of microbiota communities. However, antibiotics reduce the diversity of the community significantly. In contrast to antibiotics, rhein modulates gut microbiota and reduces the FBG of db/db mice without decreasing the diversity of the community. It is reported that increased diversity of gut microbiota may correlate with improved metabolism. Individuals from Western cultures who have a high-fat and high-sugar diet have a lower taxonomic diversity. That could result in important microbial symbionts being lost from the broader population, possibly leading to the extinction of bacterial species that can provide important health benefits [19].

The following double-blind randomized controlled trial was performed a few years ago. Insulin-resistant males with the metabolic syndrome received either autologous or allogenic feces infusion from lean donors. Beneficial metabolic effects were observed in the group receiving the lean donor transplantation, including a significantly improved peripheral (muscle) insulin sensitivity and a significantly increased intestinal microbial diversity [20]. In our study, we demonstrated that antibiotics, in addition to decreasing the FBG, decrease the diversity of the community significantly. In contrast to the effects of antibiotics, rhein modulates gut microbiota and reduces the FBG of db/db mice without decreasing the diversity of the community. Our study also shows that mice treated with antibiotics have a significantly higher relative abundance of proteobacteria than other bacteria. The gut flora in mice gavaged by rhein + antibiotics is similar to mice treated with antibiotics alone. Alterations in the gut microbiota are associated with metabolic disorders [20]. The origin of the metabolic endotoxemia could be linked to an increased proliferation of Gram-negative bacteria, notably Proteobacteria. The first randomized controlled trial involving FMT for relapsing CDI (Clostridium Difficile Infection) demonstrates vast superiority of FMT (Fecal Microbiota Transplantation) over traditional antibiotic therapy. After FMT, there is increased bacterial diversity, with increases in Bacteroidetes species and Clostridia classes IV and XIVa and decreased Proteobacteria [21]. Recent data show that increased Proteobacteria, Enterobacteriaceae, and* Escherichia* are the only phylum, family, and genus types exhibiting significant differences between obese and NASH microbiomes [22]. A compensatory increase in genera from the Proteobacteria phylum is also observed in antibiotics-treated nonobese diabetic (NOD) mice that spontaneously develop autoimmune type 1 diabetes [19].

Additionally, we present evidence that rhein elevates plasma GLP-1 level in db/db mice. L-cell immunohistochemical staining in the terminal ileum is also increased in rhein-treated mice. Enteroendocrine cells secrete a vast number of peptides with profound effects on host physiology, which has been extensively reviewed elsewhere. The most studied peptides are the glucagon-like peptide 1 (GLP-1) and peptide YY (PYY). Both are secreted by L-cells that are most abundant in the distal small intestine and have several biological functions in host physiology ranging from controlling appetite and regulating stomach emptying and gut transit to acting as incretin hormones and promoting b-cell survival and proliferation [5]. Enhanced L-cell secretion would have the potential local peptide effects in the gut and distant hormonal actions in the portal venous system and liver. Emerging evidence suggests that GLP-1 is involved in the regulation of energy balance and glucose homeostasis via the digestion of specific dietary fibers or nondigestible carbohydrates by gut microbiota fermentation [21]. The major products of nondigestible carbohydrate fermented by the gut microbiota are short-chain fatty acids (SCFA; e.g., acetate, propionate, butyrate, lactate, and succinate), which are able to stimulate gut peptide production. The SCFA is reported to stimulate GLP-1 and PYY secretion via G-protein-coupled receptor FFAR2/GPR43 [22].

## 5. Conclusions

Taken together, our study shows that oral administration of rhein can significantly reduce the level of FBG and elevate GLP-1 in db/db mice. However, the antidiabetic effects of rhein can be abrogated in db/db mice if they are given broad-spectrum antibiotics before rhein treatment, although the antibiotics alone have a hypoglycemic effect. Furthermore, rhein treatment maintains the diversity of microbiota communities, while the antibiotics reduce the diversity of the community significantly. In the present study, the abundance of* Bacteroides* and* Akkermansia* is increased in mice treated with rhein. Our study provides circumstantial evidence that gut microbiota might be involved in the antidiabetic effect of rhein, although the specific mechanism is still unclear. Clinical trials should be performed to further assess the possibility of applying rhein to diabetic patients.

## Data Availability

The data used to support the findings of this study are available from the corresponding author upon request.
